# Developing a consensus-driven, plain-language clinical research glossary for study participants and the clinical research community

**DOI:** 10.1017/cts.2022.12

**Published:** 2022-01-31

**Authors:** Sylvia Baedorf Kassis, Sarah A. White, Barbara E. Bierer

**Affiliations:** 1 Multi-Regional Clinical Trials Center of Brigham and Women’s Hospital and Harvard, Cambridge, MA, USA; 2 Division of Global Health Equity, Department of Medicine, Brigham and Women’s Hospital, Boston, MA, USA; 3 Department of Medicine, Harvard Medical School, Boston, MA, USA

**Keywords:** Clinical research, health literacy, plain language, glossary, patient-centricity

## Abstract

Clinical research is complex, and research-related terms can be challenging to understand. Clear, supportive communication with patients, potential study participants, and their caregivers must be prioritized by healthcare providers as well as investigators and their research teams. In clinical research, health literacy best practices support the ethical tenets of respect, justice, and beneficence. Plain language advances the understanding of informed consent documents, as well as comprehension of educational information, recruitment materials, study instructions, and study results summaries, among others. Further, a more collaborative research partnership is fostered when study participants are given understandable materials, while a lack of understanding can delay accrual and decrease adherence. We launched a pilot initiative to develop a consensus-driven, plain language clinical research glossary to promote clarity, consistency, and transparency across clinical research stakeholder groups. The resulting resource, described herein, is intended to be used widely to support a greater understanding of clinical research and empower study participants. Considerations for expansion are also discussed.

## Background and Introduction

Clinical research is essential for the discovery of medical interventions that advance public health and medicine. Scientific concepts, including research interventions, procedures, and instructions, are often complicated to explain to people who are not familiar with medicine and clinical research. Even individuals with advanced education can struggle to make sense of unfamiliar and technical content related to a medical condition or intervention when deciding whether to participate in a research study.

Many government agencies, life sciences companies, health systems, academic institutions, nonprofit organizations, insurers/payors, foundations, and others have developed health-related and disease-specific glossaries. Several of these glossaries [1,2] have been designed to meet the specific needs of a more technical audience of scientific stakeholders but not necessarily those of nonscientists. As a result, definitions for complex terms often use other complex terms. Further, even glossaries developed for use by the general public and patients are frequently more focused on medicine and health concepts [3,4] than on research. The United States Food and Drug Administration (FDA) and European Medicines Agency (EMA) both endorse plain language in medicine and clinical research [5,6], but few resources exist to support the use of plain language in research and even fewer have involved patients or participants in their development.

Serving as a neutral convener of representative stakeholders from across the clinical research industry, the Multi-Regional Clinical Trials Center of Brigham and Women’s Hospital and Harvard (MRCT Center) previously led a workgroup to develop a website of resources [7] to support the integration of health literacy best practices into clinical research [8]. Plain language is a cornerstone of health literacy and involves writing in a way that helps all people understand the material, without unneeded words or technical jargon. Over the course of the health literacy project, the workgroup found that a comprehensive and publicly available plain language glossary of clinical research terms and procedures co-created with patients, caregivers, and other clinical research industry stakeholders did not exist. Research terms and their definitions and uses are often technical and cover a wide range of activities, from the time potential study participants first learn about research and are recruited to a study, through the informed consent process, study procedures, to the end of the study when study results may be shared. As a result, the workgroup suggested that a common clinical research glossary applicable across all participant-facing clinical research communications, annotated with supplementary information, graphics, videos, and other related resources be developed to help study participants and their caregivers understand the process and encourage consistency in word choice by content developers. In addition, a general clinical research glossary might be useful to the broader public, and support access to clinical research.

In 2020, the MRCT Center initiated a pilot project to develop a collaborative, consensus-driven, plain language clinical research glossary [9]. The pilot process led to the development of a web-based, proof-of-concept^1^ Clinical Research Glossary website that is publicly available for use [10]. Importantly, the Clinical Research Glossary is designed to be accessed and used by patients, current and future study participants, and their caregivers directly. Here, we describe the utility, process, and limitations of developing the resource, as well as considerations for future expansion.

## Approach

The goal of this pilot was to develop a process to co-create plain language definitions of clinical research words by using a multi-stage, collaborative, consensus-building approach. To that end, the initiative had two aims: (1) developing and reviewing content and (2) establishing a group consensus-building process to arrive at agreed-upon plain language definitions.

### Development

The 53 American English terms were selected by MRCT Center leadership (SBK, BEB, SAW) to encompass a broad mix of frequently used terms based on a qualitative review of a variety of participant-facing materials deployed in clinical research (including recruitment and informed consent documents and plain language summaries). The proposed list was further cross-referenced against an existing glossary of controlled terminology for research [2] and existing patient-facing glossaries such as Just Plain Clear® [3] to select words that were not already defined in plain language. The final list comprised those that would be likely to appear at different points in a participant’s clinical trial journey. Nonetheless, given that this was a pilot project, we were not overly discriminating as we expected the glossary, if utilized, to be expanded.

An initial prototype plain language definition for each term was then developed (SBK, BEB) using health literacy principles [e.g. plain language, words with few syllables, active voice, shortened sentences, fewer ideas in a single definition, fewer idioms and jargon, and removal of additional, non-essential information that could be shared in different sections of the word’s webpage (such as, the use in context or additional info)]. The definition was iterated upon until agreeing on an initial definition that was sent to the Clinical Research Glossary Workgroup.

**Fig. 1.**
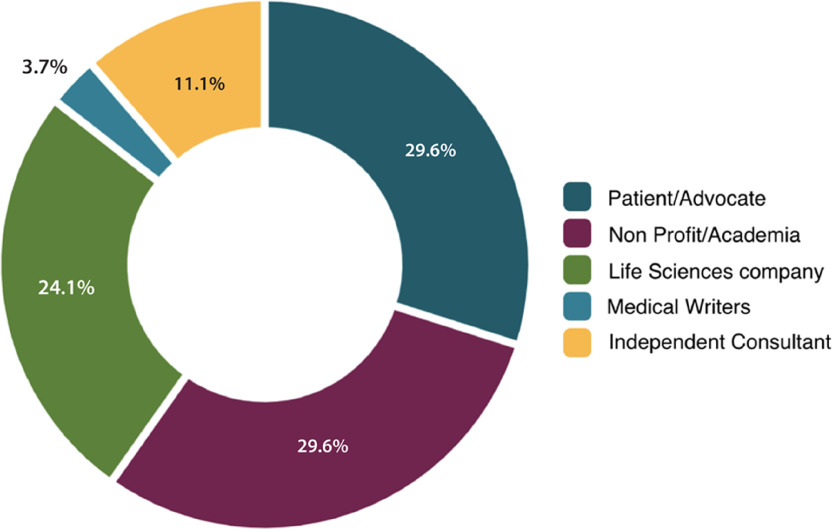
Categorical representation of different stakeholder groups on the Clinical Research Glossary workgroup to ensure broad inclusion of diverse perspectives. Workgroup members self-defined among given categories.

The Clinical Research Glossary Workgroup volunteers (n = 27) were identified through professional contacts based on their professional roles, personal experiences, or involvement in clinical research that would allow them to offer diverse perspectives on the words, definitions, and their uses in research-related communications. All identified individuals who were interested in participating were selected. In order to solicit a broad mix of perspectives, the workgroup included patients and advocates (29.6%, n = 8), as well as individuals from academia/non-profit organizations (29.6%, n = 8), life sciences companies (24.1%, n = 7), medical writers (3.7%, n = 1), and independent consultants (11.1%, n = 3), (See Fig. 1). All workgroup members had some familiarity with clinical research and/or developing clinical research communications for an audience of patients/participants. The workgroup also included international English speakers: one person from the United Kingdom, one from Australia, two from Canada, and one each from France and Germany who spoke English as a second language. The patient/advocate representatives reflected a wide range of ages and conditions, including an adolescent and her parent. Further, every workgroup meeting included at least 25% patient/advocate representation.

### Review

Feedback on the definitions was collected over 6 months using an agile [11] process. Typically applied to software development, an agile process is a way to manage a project in several discrete phases or sprints [11]. It emphasizes collaboration among stakeholders, encouraging continuous process improvement and responsiveness to change at every stage [12]. It was important to develop a methodology in this pilot project that would be efficient and sustainable in future expansion.

It should be noted that an agile process is not a consensus-building methodology. The agile process depends upon multiple *a priori* planned check-in points to allow and encourage modifications and improvement in methods by members of a team. An agile approach, however, does share some similarities with other, more formalized consensus-building methodologies. Like other consensus methods (e.g., Delphi), the agile process depends on heterogeneity of participants and allows for multiple rounds to develop a consensus definition [13]. The agile process does include collaboration and discussion, and there is no effort to maintain anonymity among the participants.

Workgroup volunteers self-selected into one of two groups: the Development Team or the Review Team. Though not required, each member remained in whatever team they had selected for the duration of the pilot. Members were provided with background and information on health literacy and its importance in clinical research, and the agile methodology, both in one-on-one introductory meetings with MRCT Center staff (SBK) and initial workgroup meetings to kick-off the pilot.

Each of the four sprints consisted of up to 10 days for the Development Team to provide input, approximately 3 days for the MRCT Center (SBK, SAW, BEB) to integrate feedback, and then approximately 7 days for the Review Team to evaluate the updated definitions. Each sprint included 10–15 terms, their definitions, and a decision-making process to determine whether any definitions could be finalized. The pilot included regular all-team member meetings for questions, to review the process, and to discuss progress.

To facilitate a sprint process that allowed multiple individuals to provide feedback simultaneously, Google Sheets was used to circulate definitions electronically for workgroup members to review in each sprint. This cloud-based method of written feedback collection allowed simultaneous review and documentation of different perspectives that were then used to iterate on the original definitions without compromising efficiency. During each sprint cycle, the Development Team commented on the initial proposed definition with suggested revisions plus their rationale for any changes the MRCT Center should consider before sending updated definitions to the Review Team. The Review Team then evaluated these definitions, deciding whether each was acceptable in its current form and, if not, identifying the area(s) of concern.

**Fig. 2.**
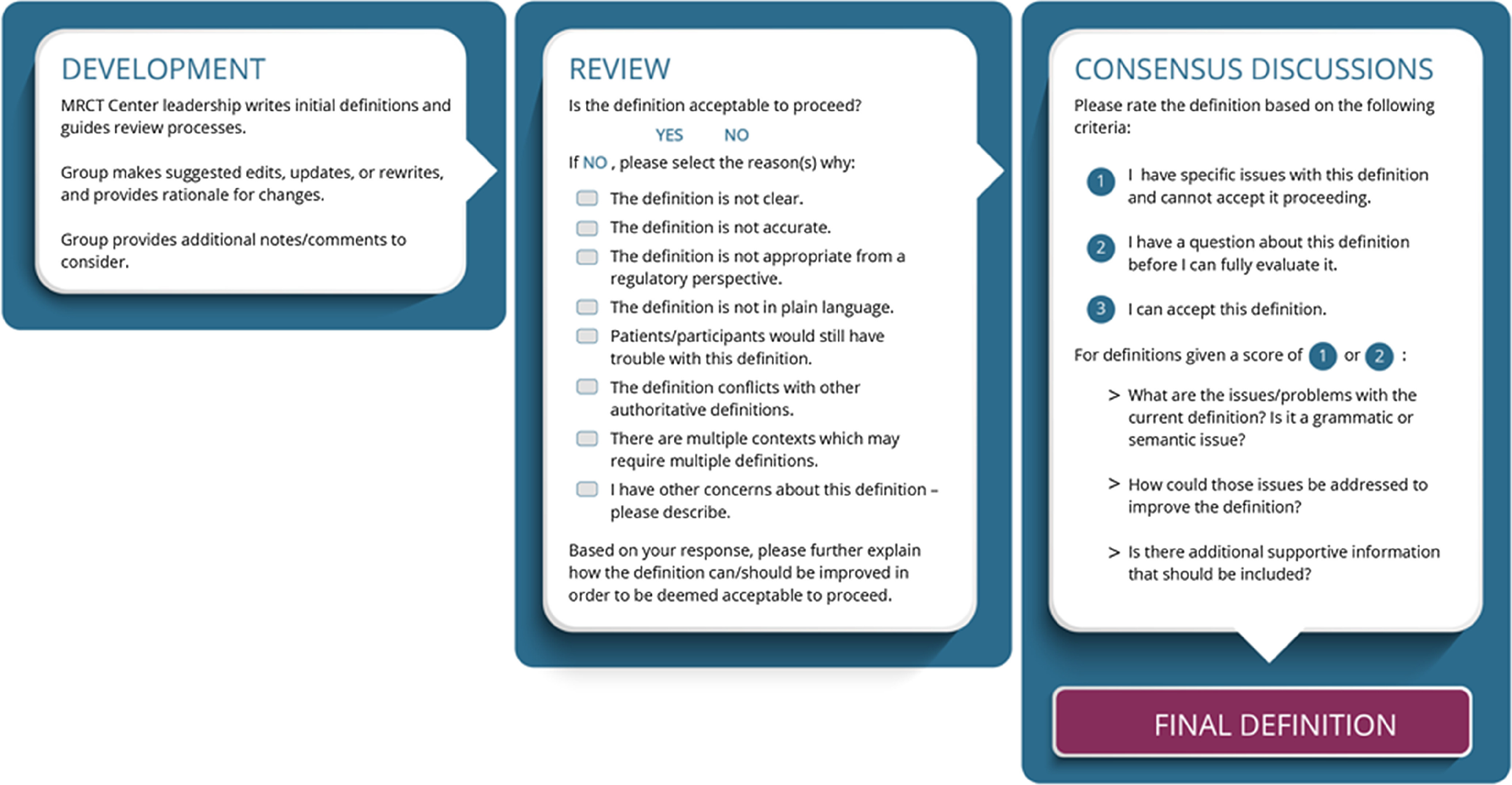
Workgroup definition development, review criteria, and process workflow during the conduct of the Clinical Research Glossary pilot. Workgroup members evaluated definitions to refine the definitions iteratively and until consensus was achieved.

Review criteria were co-created with workgroup feedback and evolved over the course of the pilot. The definition of each term was evaluated across eight review criteria: (1) clarity, (2) accuracy, (3) consistency with regulatory definitions, (4) use of plain language, (5) understandability to the patient/participant, (6) concordance with other authoritative (e.g., Clinical Data Interchange Standards Consortium – CDISC) [2] definitions, (7) use across contexts, and (8) other concerns (Fig. 2).

Twelve of the 53 terms (clinical benefit, clinical research, confidentiality, discontinue, withdraw, enroll, healthy volunteer, study participant, multicenter trial, baseline assessment, data, and protocol) reached an agreed-upon plain language definition within a single sprint cycle and did not require discussion. These words were generally considered relatively easy to define and non-controversial. Any written comments were readily resolved. Notably, while patients/advocates had been included from the beginning of the pilot process, the MRCT Center team brought patients/advocates from both the Development and Review Teams together for an additional opportunity to review and discuss these twelve definitions via separate virtual meetings. It was important to ensure that patients/advocates felt encouraged to provide their perspectives and not be inhibited by the larger academic/technical group. Based on these conversations, a few additional, patient-identified updates were made, re-reviewed by the workgroup, and finalized.

### Consensus Discussions

The remaining 41 plain language definitions could not be finalized via cloud-based written feedback alone within a single sprint cycle. Development and Review Team members then met weekly for 2-hour, facilitated virtual meetings over 5 weeks until consensus was achieved. These conversations permitted workgroup members to explain and clarify different perspectives and elements of confusion or imprecision (such as, where definitions contained grammatical inconsistencies in a part of speech between the term and the definition, defined a term only by using another in the glossary and vice versa, might be difficult to translate to another language because of dependency upon idioms, were insensitive to people with disabilities, or were potentially disrespectful). For example, the group decided that the use of blind/blinded/blinding to describe the experience of individual study participants (e.g., “the study participant was blinded”) was not appropriate or respectful, yet the term would be suitable in the context of the study design (e.g., “the study participant enrolled in a single-blind study”). During these meetings, viewpoints were solicited and shared; an MRCT Center moderator (SBK) summarized key points. Consensus was reached when no additional concerns were raised, and all workgroup members were comfortable with proceeding.

The consensus discussion criteria focused on whether workgroup members could accept a final definition (Fig. 2). All definitions that were included in the final pilot glossary were deemed acceptable by the group. The consensus approach utilized within the context of this pilot was a practical method that was created by the group and could be used beyond the pilot if the glossary were to be expanded.

How certain definitions evolved during the pilot was instructive. First, only one word, “placebo,” failed to achieve consensus. The workgroup was unable to agree on a broad plain language definition given its use in different circumstances. For example, rather than agree on the proposed definition of placebo as an inactive mimic of the active study intervention, specific examples such as sham surgery, a device that is implanted but not activated, or a medicine that looks like the interventional product but has no active ingredients were proffered. Given its complexity the group elected to revisit this word in the future. Second, one initial word, “blinded,” evolved into two separate words, “single-blind study” and “double-blind study.” As noted above, the group endorsed person-first language [14–16], rejected the notion that study participants are “blinded,” and focused on the study design element of single-blinded versus double-blinded studies. Third, the workgroup added one term, “outcome measure.” During discussion, the workgroup felt defining “outcome measure” was a natural extension of defining “outcome” and should be added to the glossary. Please see Table 1 for examples of how three definitions evolved through review and consensus conversations. After all plain language definitions were finalized, and the standard template for definitions, supplemental information, and the overall Clinical Research Glossary website interface were developed.

**Table 1.** Examples of definition evolution

Word	Initial definition	Updated definition	Final definition	Critical feedback
Efficacy	How well the study treatment causes the intended effect in a study population	A measure of how well a study treatment works and if it has the effect the researchers expected	How well a study treatment works in the study	– Keep the definition short and focused on a single concept (i.e. remove the information on researcher expectations)
Pharmacokinetic study	A way to measure how a drug travels through a person’s body over time	A way to measure how a drug is processed by a person’s body over time	A study that measures what happens to a drug in a person’s body over time	– Avoid adding confusing information (i.e. drug traveling) and simplify
Randomization	A way to enroll study participants into different study arms that does not involve the researcher or study participant choosing what intervention they get	A method for study participants to get into different groups by chance	A way to use chance to place study participants into different study treatment groups	– Focus on a single idea (i.e the use of chance for a study assignment) and simplify so that the subject of the sentence isn’t confusing (i.e. unclear who ‘they’ was referring to)

**Fig. 3.**
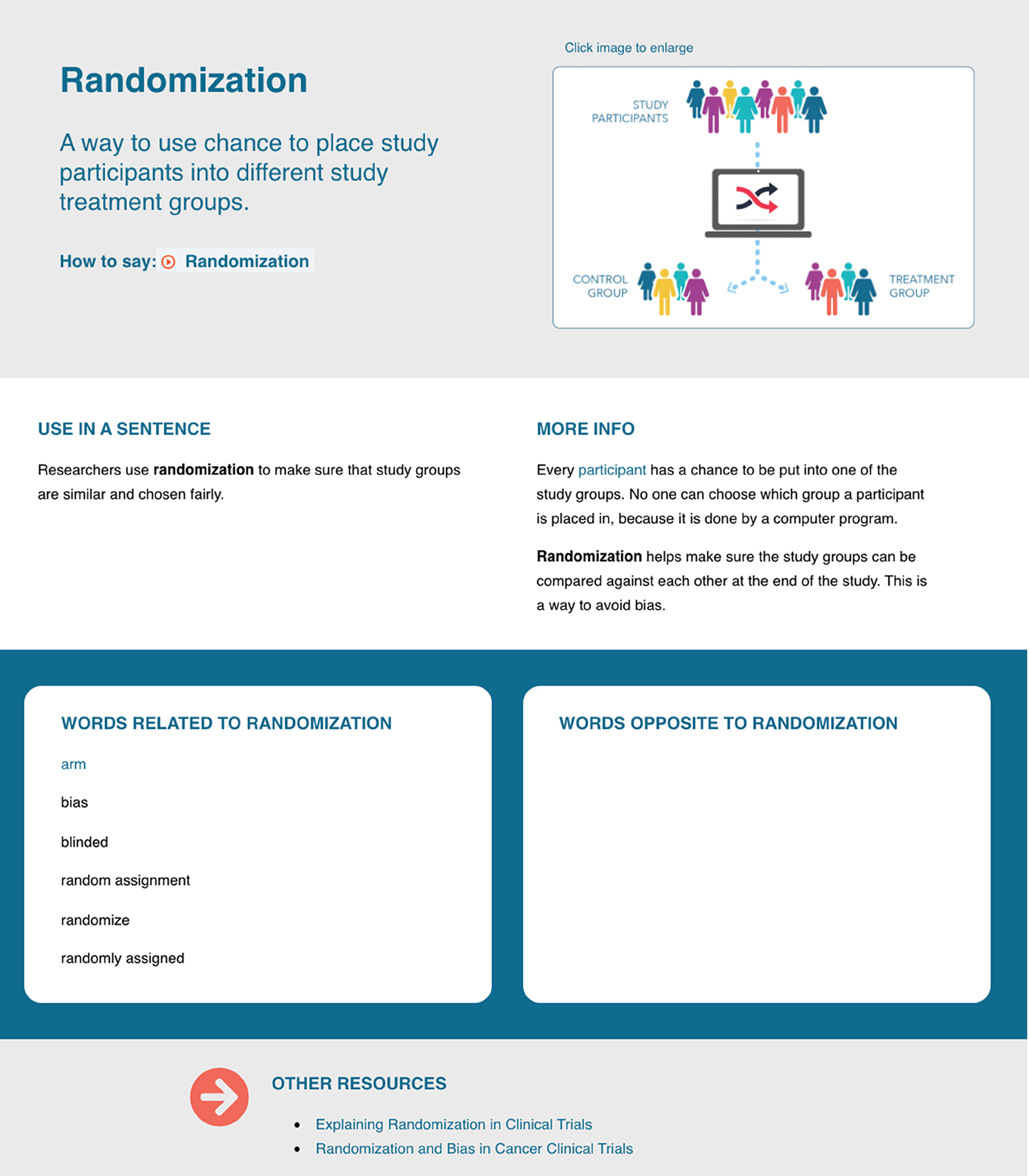
Example of a clinical research word, plain language definition, and additional elements that are included for each term.

The template format for each term included the word or term itself, followed by a plain language definition. The supplemental information included six additional elements: (1) a supporting image or graphic, (2) use in a sentence, (3) more information, (4) related words, (5) opposite words, and (6) other resources that could provide additional context for the definition (see Fig. 3 for an example, and www.mrctcenter.org/clinical-research-glossary for the complete pilot glossary).

### Additional Feedback

The standard template of elements and their adaptation to the web were reviewed for health literacy best practices, including plain language, user-friendly design elements, and end-user feedback collection. Usability testing included review of words and definitions by patient/advocate reviewers and/or project-naïve individuals and focused on the reviewers’ endorsement of the understandability of the definition and acceptability/functionality of the site.

The self-identified patient/advocate reviewers (n = 5) were members of the workgroup or project-naïve patient representatives who were recruited through a local Patient and Family Advisory Council (PFAC) and provided anonymous feedback on content and web design. Additional reviewers (n = 3) included individuals affiliated with the MRCT Center but not familiar with the pilot. All reviewers represented a subset of potential users of the glossary website. They were invited to answer several questions related to content and functionality (further captured in Table 2).

**Table 2.** Solicited feedback on glossary content and website from additional reviewers

Review focus	Feedback solicited
**Content**	• Do you have any questions, or is there anything unclear, about any of the plain language definitions you reviewed? • If there is a graphic, does it match the word and definition? Would you suggest any changes? • If there is no graphic, do you have any suggestions on what kind of graphic should be added? • Are the other fields of text information clear? Would you update/change any of it? • Are there any other resources you would recommend we link to?
**Website design and layout**	• Describe the purpose of the site and the intended audience. • Navigate through the site and describe your experience using it. • Randomly select three words and rate the definition and image/graphic. • Comment on the ease of using the site, the likelihood of using it in the future, and the likelihood of recommending it.

Before launch, all workgroup members were given another opportunity to provide feedback on the content, layout, and design of the site.

## Discussion

The Clinical Research Glossary pilot represents a collaborative, multi-stakeholder effort to develop a common resource to support patients’, study participants’, and the public’s understanding of clinical research. The experience of developing individual plain language definitions and the larger website offered several observations regarding the process of consensus-building with a multi-stakeholder, patient-engaged group, the possible expansion of this effort in the future, and limitations of this work.

### Process Learnings and Expansion Considerations

A primary design element of the pilot was appropriate workgroup representation reflecting the research ecosystem and, importantly, including patients and patient advocates as co-creators. Broad representation of workgroup members, from groups such as medical writing, regulatory affairs, patient/advocate, life sciences companies, academia, and nonprofit organizations in the research space, helped reach accurate and balanced definitions that are understandable to a nonscientific audience yet remain consistent with their technical use in research.

The depth of discussion from differing perspectives revealed common, but unanticipated, perceptions and confusions. In addition to the issues related to the use of term “blinded” to describe study participants, the term “clinical trial” was heard as or interpreted to be “criminal trial,” a concept far more familiar in the common lexicon. Thus, replacement of the word “trial” with “study” was preferred and considered more likely to be understood as intended in the context of clinical research, as opposed to a legal proceeding in a court of law.

Feedback from usability testing led to an appreciation of the range of potential preferences not only for the definitions themselves, but also for the visual layout of all the content on the website. For example, the order of information presentation was informed by and modified based on the patient experience of using the website. As a result, future expansion will broaden the cultural diversity of workgroup membership and those recruited to perform additional project-naïve reviews to ensure the evolving clinical research glossary is accessible to, and used by, the greatest number of researchers, patients and potential study participants, and the public.

Designing the pilot to apply an agile development process was recommended by workgroup members. By setting the expectation that the process would evolve in response to workgroup feedback, the team anticipated adaptation to optimize the workflow over time. The concept of co-creation also supported the review criteria’s evolution and the elements to be included beyond the primary definition. A significant adaptation was the addition of consensus-building virtual meetings to discuss areas of disagreement in interpretation and understanding. Through these consensus conversations, for example, the group was able to resolve the definitions of term that have regulatory relevance but may not have practical significance to study participants (e.g., the difference between “adverse event,” “adverse reaction,” and “side effect,” which for study participants would generally be considered “bad things that might happen in the study”). As a discrete pilot of a predetermined number of term, consensus conversations were held at the end, after all sprints were complete. Glossary expansion efforts, however, with a larger number of words, will build consensus-building discussions into each sprint for a more direct and efficient path towards an agreed-upon definition on a regular and predictable timetable. Agile flexible adaptation will continue to be an important aspect as future expansion is considered.

An unexpected learning was the need to modify the accessibility of the process itself: workgroup members, some of whom have chronic illness or disability, expressed their need for adaptations to support their participation. Those changes included increasing the font size of written materials and closed captioning during discussions. While some modifications (e.g., offering written materials with an option to change font size) can be anticipated and prepared in advance, others, such as the need for a transcript of live captions or a 1-on-1 session to accommodate slow speaking rate, may arise during the process. The discovery and solutions for such accommodations are facilitated by clear communication and the agile process.

Consistent workgroup member meeting attendance built rapport and supported the acceptance of diverse opinions. Recognizing and addressing differences in understanding reflected a primary goal of the work itself. The diversity of the workgroup and the recognition of differences substantiated the need for the glossary and allowed the team to focus on editing definitions of greater nuance or substance. Further, the familiarity with one another over time resulted in efficiency and respect. Any expansion effort will again identify a consistent group of people to work towards the common goal.

In addition to expanding the number of term and procedures, the agile process allows new words to be ‘cycled’ in. Sprints can accommodate additions (through public engagement, suggestions from workgroup members, or user feedback) to ensure an expanded and evolving glossary includes timely and relevant information (e.g., “emergency use authorization,” during the COVID-19 pandemic). The process also promotes dynamic modifications of definitions if additional complexities or uses arise.

The MRCT Center plans to expand the glossary and has forged organizational partnerships to select additional words, develop new content, build consensus with a continuation of a workgroup comprised of former and new members, and establish a more sustainable platform for all the information to be maintained. Dissemination activities continue through community outreach activities, seminars, conferences, and the gray literature (e.g. blog posts and other articles).

### Limitations

Limitations of this glossary include the limited size of the pilot and that it is currently in American English only. There could be additional cultural differences in the interpretation of words that extend beyond our workgroup to other English-speaking contexts (and then, of course, to different languages as well). Inclusion of additional national and international perspectives in expansion efforts as well as review by representatives of diverse communities within and outside the USA will be prioritized.

## Conclusion

We describe the process of developing a proof-of-concept plain language clinical research glossary involving diverse stakeholders from across the patient/advocate and research communities. Plain language definitions and other relevant information were achieved by the multi-stakeholder group through an efficient, deliberate, and respectful process.

The impact of the glossary will be realized if patients, study participants and their caregivers, and the public are provided with a unified source of clinical research definitions and consistent usage of those terms. This Clinical Research Glossary offers a start at harmonization. Where avoidable, terms should not be defined differently by different organizations. Commitment across the research industry (including life science companies, academia, nonprofit organizations, patient advocacy groups and government agencies) to use common, consensus-derived words, and definitions in public-facing clinical research communications, will enhance comprehension across professional networks and among patient and study participant communities. Expansion opportunities are being explored.

## Footnotes

1 “Proof of concept” refers to the processes used to develop the definitions, additional content for each word, and the website itself, all of which were undertaken to ensure that the proposed methods for the development of the resource were feasible and resulted in an acceptable and useable product. The website is a pilot, and it is not complete. We plan on extending the number of terms in the glossary over the next years.
